# The outbreak of COVID-19 in Mulhouse: Hospital crisis management and deployment of military hospital during the outbreak of COVID-19 in Mulhouse, France

**DOI:** 10.1186/s13613-020-00677-5

**Published:** 2020-05-19

**Authors:** Khaldoun Kuteifan, Pierre Pasquier, Christian Meyer, Jacques Escarment, Odile Theissen

**Affiliations:** 1 Service de Réanimation Médicale GHRMSA, Hôpital Emile Muller, Mulhouse, France; 2 Département d’anesthésie-réanimation, Hôpital d’instruction des armées, Clamart, France; 3 Ecole du Val-de-Grâce, Paris, France; 4 Elément Militaire de Réanimation du Service de Santé des Armées, Mulhouse, France; 5 Service de Réanimation Chirurgicale, GHRMSA, Hôpital Emile Muller, Mulhouse, France; 6 Service d’Anesthésie, GHRMSA, Hôpital Emile Muller, Mulhouse, France

The outbreak of a novel coronavirus in Wuhan, the capital city of Hubei, China, in December 2019 raised a worldwide concern. Cases of pneumonia of unknown origin were first described and the pathogen responsible was later identified as being a novel betacoronavirus [1]. Named as the Severe Acute Respiratory Syndrome coronavirus-2 (SARS-CoV-2), this virus is responsible of the coronavirus disease 2019 (COVID-19) [2]. The outbreak began in the east of France during the first days of March after a religious meeting gathering about 2000 people, which took place in Mulhouse from 17th to 24th February 2020.

The *Groupe Hospitalier de la Region de Mulhouse Sud*-*Alsace* (GHRMSA) is a hospital complex including 10 sites with a total capacity of 2612 beds and covering the needs of 480,000 people. Intensive care facilities are located on the main site, Emile Muller Hospital, with a capacity of 824 general beds and 2 ICUs, one medical of 20 beds and one surgical of 16 beds. The first patient presenting with respiratory failure was admitted to medical ICU on March 2nd, 2020. After 4 days, 10 patients with COVID-19 were admitted. Non-COVID-19 patients were transferred from medical to surgical ICU in the first week in order to increase ICU surge capacity for COVID-19 patients. In the beginning of the second week, all medical ICU beds were occupied by COVID-19 patients. The cardiac surgery ICU (4 beds) was transformed to a COVID-19 ICU, as scheduled surgical and medical activity was stopped. Sixteen additional beds were added in five operative and post-operative rooms. Eight beds “non-COVID-19” ICU was created in another post-operative room. Surgical ICU was also converted into COVID-19 ICU.

On week 3 of the outbreak, in an unprecedented manner in France, a 30-bed field military intensive care hospital was deployed on the hospital car park by the French military medical service: the *Élément Militaire de Réanimation du Service de Santé des Armées* (EMRSSA).

So as to cope with this massive flood, a national as well as an international cooperation was setup and many patients were transferred to receptive ICUs outside the region via Alsace Regional Health Agency coordinating office, assessed by a military coordinating office, by helicopter, trains and military aircrafts in France, Germany, Luxembourg and Switzerland.

From March 2nd to April 26th, 2020, 1249 COVID-19 patients were admitted to the hospital. Among them, 278 were hospitalized in ICU. Forty-four patients were discharged from ICU, 118 were transferred to another ICU, and 42 are died. The field military intensive care hospital admitted 47 patients during the same period.

Surges in the number of critically ill patients with COVID-19 had occurred rapidly. Thus, ICU practitioners and hospital administrators had to adapt the capacity of ICU beds in number, and also in equipment, consumables, pharmaceuticals, and staffing. The staff was composed of intensivists and anesthesiologist-intensivists with one intensivist in charge of patients’ orientation every day. Nursing team was composed of ICU nurses and nurse anesthetists.

The EMRSSA mission aimed to achieve two main objectives under strenuous conditions: first to treat critically ill patients with COVID-19-associated acute respiratory distress syndrome (ARDS) according to the best standards of care; and second, to protect the caregivers. In the first 3 weeks, more than 40 critically ill patients with COVID-19 were transferred into the EMRSSA field hospital. All of them presented under ARDS conditions and required initial protective mechanical ventilation. Each ICU wing was staffed by one anesthesiologist-intensivist, three nurses (including one certified registered nurse anesthetist), and three assistant nurses, coming from the eight military training hospitals, following European recommendations on structural and organizational requirements for ICUs [3].

The protection of the EMRSSA personnel was one of the two main objectives of the mission. As a consequence, with the help of an infection control team, who experienced Ebola crisis management in Guinea in 2015, the EMRSSA was separated into three different zones according to three different protection levels: a green zone (wearing of surgical mask); an orange zone (wearing of FFP2 mask): and a red zone (wearing of full personal protective equipment: FFP2 or FFP3 mask, gown, gloves, eye protection, and apron) [4].

Most countries cannot match China’s feat of rapidly building new hospitals and ICUs during the COVID-19 outbreak in Wuhan [5]. The government decision to deploy the military hospital was more adapted to our reality. Its contribution was crucial in the face of a challenge that progressed with each day. Mulhouse hospital ICU staff and military hospital staff worked together to handle the outbreak.

## Data Availability

Not applied.
